# METTL1-regulated tRF-5001b suppresses lung adenocarcinoma by impairing QARS1 interaction with tRNA^Gln^ and inhibiting HMGA2 translation

**DOI:** 10.1016/j.ncrna.2026.09.003

**Published:** 2026-11-03

**Authors:** Jingyuan Wang, Fan Wu, Yanli Li, Zhiyu Wu, Qiyue Song, Weiwei Wang, Qiangling Sun, Zhongliang Ma

**Affiliations:** a Lab for Noncoding RNA & Cancer, School of Life Sciences, Shanghai University, 381 Nanchen Road, Shanghai, 200444, China; b Department of Thoracic Surgery, The Third Affiliated Hospital of Kunming Medical University, Yunnan Cancer Hospital, Yunnan Cancer Center, Kunming, 650118, China; c Institute of Thoracic Oncology, Shanghai Chest Hospital, School of Medicine, Shanghai Jiao Tong University, Shanghai, 200030, China

**Keywords:** Lung adenocarcinoma, tRF-5001b, QARS1, Translation, METTL1, HMGA2

## Abstract

**Background:**

Lung adenocarcinoma (LUAD) remains the leading cause of cancer-related mortality worldwide, yet the impacts of tRNA-derived fragments (tRFs) on its progression are not fully understood. This study aimed to investigate the role of tRF-5001b in LUAD.

**Methods:**

The expression of tRF-5001b in LUAD tissues was analyzed using public datasets and validated by Northern blotting. The biological functions of tRF-5001b, QARS1, and HMGA2 were evaluated using EdU, transwell, and CCK-8 assays in vitro, as well as subcutaneous and intravenous injection models in vivo. RNA fluorescence in situ hybridization and immunofluorescence were performed to determine the subcellular localization of tRF-5001b and QARS1. The expression levels of METTL1, WDR4, QARS1, and HMGA2 were examined by qRT-PCR and Western blotting. RNA immunoprecipitation, RNA pull-down, silver staining, co-immunoprecipitation, and molecular docking were conducted to investigate the interaction between tRF-5001b and QARS1. Puromycin incorporation assays were performed to assess the effect of tRF-5001b on global protein translation.

**Results:**

tRF-5001b was significantly downregulated in LUAD tissues and exhibited tumor-suppressive effects by inhibiting tumor cell proliferation, migration, and invasion both in vitro and in vivo. Mechanistically, tRF-5001b competitively disrupted the interaction between QARS1 and tRNA^Gln^, thereby suppressing HMGA2 translation and attenuating LUAD progression. Furthermore, METTL1 was identified as a negative regulator of tRF-5001b biogenesis through catalyzing m^7^G modification of tRNA-Tyr-GTA.

**Conclusion:**

This study identifies tRF-5001b as a suppressor of LUAD progression through inhibition of HMGA2 translation and highlights METTL1 as a key regulator of its biogenesis. These findings provide new insights into tRF-mediated molecular mechanisms and suggest potential therapeutic targets for lung adenocarcinoma.

## 1 Introduction

With the improvement of people's health awareness and the development of targeted therapy and immunotherapy, the morbidity and mortality rates of lung cancer are decreasing year by year, and the prognosis of early lung cancer tends to be favorable [1]. However, lung cancer is still the cancer with the highest mortality rate in the world [1]. Among the world, the majority of clinically diagnosed lung cancer cases are non-small cell lung cancers, and lung adenocarcinoma (LUAD) is the most common histologic subtype, which has a poor prognosis due to its lack of early symptoms and high rate of metastasis [2], [3]. Given these clinical challenges, there is an urgent need to identify novel molecular regulators that contribute to LUAD progression and may serve as potential diagnostic biomarkers or therapeutic targets.

tRNA-derived fragments (tRFs), 14-50 nucleotides (nt) in length, originate from the precise cleavage of tRNA-specific sites by nucleic acid endonucleases. Based on length, sequence and cleavage site of tRNAs, tRFs are broadly categorized into four groups, including tRF-5s, tRF-3s, tRF-1s and i-tRFs. tRFs are derived from mature tRNA in the cytoplasm, except for tRF-1s generated from the 3′ end of the precursor tRNA in the nucleus [4], [5], [6]. It has been shown that tRF is involved in a variety of molecular regulatory mechanisms, in order to perform biological functions that regulate gene transcription, protein translation, and epigenetic inheritance [7], [8], [9]. Recent studies have indicated that tRFs exert gene regulatory functions through miRNA-like target gene silencing in LUAD. For example, tRF-Leu-CAG was revealed that promotes cellular autophagy and chemoresistance in LUAD by targeting the 3′-UTR region of TCEA3 to silence its expression [10], [11]. In addition, tRFs affect stabilization and condensation properties of proteins through interactions that regulate the function of protein molecules to exert regulatory functions on LUAD. 5′-tRF^Cys^ induces nucleolin oligomerization to hinders the cleavage of nucleolin-bound transcripts by nucleic acid exonucleases and ultimately facilitate lung metastatic colonization of cancer cells [12]. AS-tDR-007333 promotes the expression efficiency of the oncogenic transcription factor Mediator complex subunit 29 (MED29) in LUAD by stabilizing Heat Shock Protein Family B Member 1 (HSPB1) [13]. Previous studies have shown that tRFs have a translational regulatory function [14], [15], [16]. The realization of this function may be mediated by certain translation-related complexes [15]. However, the relevance of tRFs to this conserved mechanism by which tRNAs are involved in translation is not known, and it remains unclear whether tRFs regulate LUAD through tRNAs. A recent study found that senescent neuronal cell-derived Glu-5′-tsRNA-CTC forms a complex with leucine-tRNA synthetase 2 (LaRs 2), which subsequently translocates into mitochondria to compete with mitochondrial tRNA^Leu^ (mt-tRNA^Leu^), and lead to impaired mitochondrial protein translation and damage to cristae organization [17]. This research demonstrated that tRFs competitively hinders the aminoacylation of tRNAs, providing a direct demonstrative basis for the potential correlation between tRFs and tRNAs structure. We speculated that the tRFs may retain a similar tRNA structure and inhibit the translation of certain proteins based on this molecular signature in LUAD. This molecular mechanism is not currently being explored in greater depth.

Methyltransferase-like (METTL) proteins belong to a subfamily of 7-strand methyltransferases, which catalyze the methylation of different substrate RNAs with SAM as the donor through an evolutionarily conserved S-adenosyl methionine (SAM)-binding domain [18], [19]. Methyltransferase like 1 (METTL1), the smaller isoform of the METTL family, catalyzes the N7-methylguanosine (m^7^G) of partial tRNAs variable loop by forming a methyltransferase complex with the WD repeat domain 4 (WDR4) [20], [21], [22]. It has been shown that the m^7^G modification favors the protection of tRNAs from nuclease degradation in translation as well as natural decay to facilitate translation of oncoproteins [23], [24], [25]. Furthermore, METTL1 was similarly observed in LUAD to regulate tRNA stability by mediating m^7^G modifications [26], [27]. However, whether the increased stability of tRNAs mediated by m^7^G modifications regulates tRFs biogenesis remains unknown. To date, only one study has shown that deletion of METTL1 results in loss of tRNA m^7^G modification thereby mediating the biogenesis of a series of 5′-tRFs with tumor suppressor effects [28], suggesting potential exploration of METTL1 and tRFs biogenesis. Therefore, exploring the molecular mechanisms by which METTL1 regulates tRF biogenesis is beneficial in revealing the specific association of tRFs with parent tRNAs and is a favorable argument for explaining tRFs that function as cancer suppressors in LUAD.

In the present study, we identified tRF-5001b, a previously unproven tRF, and demonstrated that tRF-5001b inhibits the development of LUAD in vitro and in vivo. Mechanistically, tRF-5001b inhibits the function of Glutaminyl-tRNA Synthetase 1 (QARS1) by competing for its tRNA binding site and inhibits its function of binding tRNA^Gln^, hindering the translation efficiency of High Mobility Group AT-hook 2 (HMGA2). Our study provided strong evidence for the development of clinical diagnostic and therapeutic programs for LUAD based on tRF-5001b.

## 2 Materials and methods

### 2.1 Tissue specimens and cell lines

Clinical lung adenocarcinoma specimens used in this study were obtained from Yunnan Cancer Hospital and were approved for academic research by the Ethics Committee of Yunnan Cancer Hospital. A total of 35 pairs of LUAD tumor tissues and matched adjacent non-tumor tissues were collected from patients. All specimens were freshly collected and immediately subjected to RNA extraction using trizol reagent following tissue collection. HEK-293T, A549, and BEAS-2B cell lines were purchased from the American Type Culture Collection (ATCC) or the Chinese Academy of Sciences. HEK-293T, A549, and BEAS-2B cells were maintained in DMEM (Gibco) supplemented with 10% fetal bovine serum (FBS; Gibco) and 1% penicillin–streptomycin (Gibco). H1299, H1975, PC-9, H1734, and H460 cells were cultivated in RPMI-1640 (Gibco) with 10% FBS and 1% penicillin–streptomycin. All cells were cultured under standard conditions at 37 °C with 5% CO_2_.

### 2.2 Animal experiments

Five-week-old female specific pathogen–free (SPF) BALB/c nude mice were obtained from Jiangsu Jicui Yaokang Biotechnology and housed in the SPF animal facility of the School of Life Sciences, Shanghai University. All animal procedures were approved by the Ethics Committee of Shanghai University.

### 2.3 Cell transfection

Cell transfection was executed using Lipofectamine 3000 Transfection Reagent (Thermo Fisher Scientific) according to the manufacturer's instructions. Briefly, 10 pM tRF-5001b mimic (RiboPharm) or 4 μg plasmid DNA was mixed with Lipofectamine 3000 and P3000 reagents in basal medium, incubated at room temperature for 15 min, and added to cells. The medium was replaced with fresh complete medium after 4–6 h for subsequent experiments.

### 2.4 Lentivirus infection

To establish LUAD cell lines with stable gene expression, lentiviral particles were generated in HEK-293T cells using Lipofectamine 3000 (Thermo Fisher Scientific). Viral supernatants were harvested at 48 h and 72 h post-transfection, cleared of cell debris by centrifugation, and added to target LUAD cells containing 8 μg/mL polybrene (Beyotime Biotechnology). Stable cells were selected using 2 μg/mL puromycin (Beyotime Biotechnology) for 48 h.

### 2.5 Cell Counting Kit-8 (CCK-8) assay

Cell viability was evaluated at 0, 24, 48, 72, and 96 h after cell seeding. At each designated time point, cells were incubated with 5% CCK-8 reagent (Yeasen Biotechnology) diluted in basal medium for 2–3 h at 37 °C in the dark. The absorbance was subsequently determined at 450 nm using a microplate reader.

### 2.6 Ethynyl deoxyuridine (EdU) assay

Cell proliferation was assessed using the EdU-594 Cell Proliferation Kit (Beyotime Biotechnology) per the manufacturer's protocol. Cells were incubated with 10 μM EdU for labeling, fixed with 4% paraformaldehyde (Beyotime Biotechnology), and permeabilized. After performing the Click reaction for EdU detection, nuclei were counterstained with Hoechst 33,342 (Beyotime Biotechnology). Images were captured under a fluorescence microscope (Zeiss).

### 2.7 Wound-healing assay

LUAD cells were seeded in 6-well plates and cultured to over 90% confluence. To eliminate the impact of proliferation, cells were pretreated with 10 μg/mL mitomycin C (Beyotime Biotechnology) for 2 h. A linear scratch was created using a 200 μL pipette tip, and wound closure was monitored and photographed at 0 h and 24 h.

### 2.8 Transwell assay

For cell migration evaluation, LUAD cells suspended in serum-free medium were introduced into the upper chambers of Transwell inserts (Corning Incorporated), while complete medium containing 10% FBS was placed in the lower chambers. For invasion assays, the upper chambers were pre-coated with Matrigel (Corning Incorporated) before cell seeding. Following incubation for 24–48 h, cells on the lower surface were fixed in 4% paraformaldehyde (Beyotime Biotechnology), stained with crystal violet (Beyotime Biotechnology), and quantified after removing non-migrated cells.

### 2.9 Quantitative real-time PCR (qRT-PCR)

Total RNA from LUAD cells or tissues was isolated using TRIzol Reagent (Thermo Fisher Scientific) via chloroform extraction and isopropanol precipitation. For small RNA detection, cDNA was synthesized using the Hifair miRNA 1st Strand cDNA Synthesis Kit (Yeasen Biotechnology). In brief, a poly(A) polymerase was first used to enzymatically add a poly(A) tail to the 3′ end of mature miRNAs and then transcribed by an oligo (dT) primer. For mRNA detection, reverse transcription was conducted using the PrimeScript RT Reagent Kit (Takara Bio). Quantitative real-time PCR was performed using Hieff qPCR SYBR Green Master Mix (Yeasen Biotechnology). U6 snRNA served as the internal control for tRNA^Gln^ and tRNA-Tyr-GTA, while GAPDH was used for METTL1, WDR4, and HMGA2. Relative RNA levels were determined via the $2^{-\Delta \Delta C_{t}}$ method.

### 2.10 tRF-5001b validation

To confirm the existence of tRF-5001b in LUAD cells, the reverse-transcribed tRF-5001b fragment was amplified by PCR and cloned into the T-Vector pMD™19 vector (Takara Bio). The ligation reaction was carried out using a DNA ligation kit (Takara Bio) at 16 °C overnight. The product was transformed into competent *E. coli* DH5α cells (Shanghai Weidi Biotechnology). Positive clones were verified by agarose gel electrophoresis and Sanger sequencing (Tsingke).

### 2.11 Western blot

Total protein was extracted using RIPA lysis buffer (Beyotime Biotechnology) supplemented with 100 mM PMSF (Beyotime Biotechnology) and quantified via a Bradford assay (Beyotime Biotechnology). Protein samples were denatured in loading buffer at 100 °C for 7 min, separated by 10% or 12.5% SDS-PAGE (Yaenzyme Biotech), and transferred to 0.45 μm PVDF membranes (Millipore). Membranes were blocked with 5% nonfat milk (Sangon Biotech) in 1× TBST (Servicebio Biotechnology) for 2 h, incubated with primary antibodies (Proteintech) at 4 °C overnight, followed by HRP-conjugated secondary antibodies (Proteintech) for 2 h. Bands were visualized using an ECL kit (Beyotime Biotechnology) and imaged on an automated gel documentation system (Bio-Rad).

### 2.12 Northern blot

Total RNA was extracted from LUAD cells using the RNAeasy™ Animal RNA Extraction Kit (Beyotime Biotechnology). Denatured RNA (≥1000 ng) was separated on 8% Urea-PAGE gels (Beyotime Biotechnology) in 1× TBE and transferred to nylon membranes (Beyotime Biotechnology) at 60 V for 1 h, followed by UV crosslinking for 7 min. Membranes were pre-hybridized at 42 °C for 2 h and hybridized overnight with a 10 nM biotin-labeled tRF-5001b probe (Sangon Biotech) at 42 °C. Membranes were then incubated with Streptavidin-HRP Conjugate (1:2000; Beyotime Biotechnology) for 15 min and visualized using BeyoECL Moon reagents (Beyotime Biotechnology) on an automated gel imaging system (Bio-Rad).

### 2.13 RNA fluorescence in situ hybridization

For RNA fluorescence in situ hybridization, LUAD cells were cultured overnight on coverslips to 30–40% confluence and fixed in 4% paraformaldehyde for 15 min at room temperature. Using an RNA FISH Kit (GenePharma), cells were permeabilized in 0.1% Buffer A for 15 min, followed by incubation in 2× Buffer C for 30 min at 37 °C. Fluorescent probes targeting tRF-5001b (2 μM; Sangon Biotech) were preheated in Buffer E for 10 min at 42 °C, applied to the cells, and hybridized overnight at 37 °C in the dark. After sequential 5-min washes at 42 °C in 0.1% Buffer F, 2× Buffer C, and 1× Buffer C, followed by a final wash in PBS (KeyGEN BioTECH), coverslips were mounted using DAPI Fluoromount-G (Sigma-Aldrich) and imaged under a fluorescence microscope (Zeiss).

### 2.14 Nuclear/cytoplasmic fractionation

For cytoplasmic and nuclear fractionation, LUAD cells were lysed in Cell Fractionation Buffer from the PARIS Kit (Invitrogen™) for 8 min on ice, followed by centrifugation at 500 × g for 4 min at 4 °C to isolate the respective fractions according to the manufacturer's instructions. The subcellular distribution of tRF-5001b was analyzed by qRT-PCR, utilizing U6 snRNA and GAPDH as nuclear and cytoplasmic RNA markers, respectively. Fractionation quality was verified by immunoblotting with antibodies against Lamin B1 and GAPDH to control for nuclear and cytoplasmic protein separation.

### 2.15 RNA pull down

To identify tRF-5001b–binding proteins, RNA pull-down assays were performed using the Pierce Magnetic RNA-Protein Pull-Down Kit (Thermo Fisher Scientific) via two approaches. For the probe-based assay, biotinylated tRF-5001b or negative control probes (BGI Genomics) were immobilized on streptavidin magnetic beads (Beaver) and incubated overnight at 4 °C with LUAD cell lysates prepared in IP lysis buffer (Beyotime Biotechnology) supplemented with PMSF and RNase inhibitor (Invitrogen). For the RNA-based assay, tRF-5001b and control RNAs (BGI Genomics) were synthesized using the T7 Quick High Yield RNA Transcription Kit (Beyotime Biotechnology), labeled at the 3′-end using the Pierce RNA 3′ End Desthiobiotinylation Kit (Thermo Fisher Scientific), and incubated with cell lysates to capture interacting proteins.

### 2.16 Silver staining and mass spectrometry

The protein samples eluted from the RNA pull-down assays were separated by SDS-PAGE on 10% polyacrylamide gels. To visualize the specific bands captured by the tRF-5001b group, the gels were processed using a Fast Silver Stain Kit (Beyotime Biotechnology) according to the manufacturer's parameters. Differential bands specific to the tRF-5001b group were excised for mass spectrometry analysis (Oebiotech).

### 2.17 Proteomic analysis

LUAD cells were collected by centrifugation, and cell pellets were instantly frozen and stored at −80 °C. Frozen samples or excised gel bands from silver staining were shipped on dry ice to Biotree Biotechnology Co., Ltd. (Shanghai, China) for liquid chromatography-tandem mass spectrometry (LC-MS/MS) analysis to screen for candidate tRF-5001b-binding proteins.

### 2.18 RNA immunoprecipitation

For RNA immunoprecipitation (RIP) assays, antibody–bead complexes were prepared by incubating Protein A/G agarose beads (Beyotime Biotechnology) with 4 μg of specific antibodies in NT2 buffer for 4 h at 4 °C. LUAD cells were lysed in RIP lysis buffer (from the BeyoRIP Kit, Beyotime Biotechnology) supplemented with DTT, RNase inhibitor, and PMSF. Clarified lysates were incubated with the antibody-conjugated beads overnight at 4 °C to capture RNA–protein complexes. After washes in NT2 buffer, complexes were eluted for 30 min at 55 °C, and the enriched RNAs were extracted and analyzed by qRT-PCR to evaluate target RNA binding and modification levels.

### 2.19 Molecular docking

Molecular docking was carried out to predict the binding affinity and interface between QARS1 and tRF-5001b. The 3D structure of human QARS1 was obtained from the AlphaFold Protein Structure Database, and the tertiary configuration of tRF-5001b was modeled using the RNAComposer server. Binding energy grids were mapped to score the binding poses, and the optimal interaction models and hydrogen bonding clusters were visualized using PyMOL software.

### 2.20 Co-immunoprecipitation

To confirm and map the exact binding regions between QARS1 and tRF-5001b, co-immunoprecipitation experiments were performed using truncated variants. Cells were lysed in 1% digitonin or 1% NP-40 buffer (Thermo Fisher Scientific) on ice for 30 min. Protein supernatants were incubated with primary antibodies against QARS1 or control IgG for 4 h, followed by the addition of Protein A/G magnetic beads (Thermo Fisher Scientific) for another 2 h at 4 °C. After four washes in washing buffer containing 0.2% detergent, samples were eluted in SDS sample buffer at 95 °C for 10 min.

### 2.21 Puromycin incorporation

For puromycin incorporation assays, LUAD cells were transfected with negative control (NC) or tRF-5001b mimics using Lipofectamine 3000 (Thermo Fisher Scientific). Forty-eight hours post-transfection, cells were incubated with 1 μM puromycin for 30 min at 37 °C. Cells were then harvested and lysed, and whole-cell lysates were separated by SDS-PAGE and immunoblotted with an anti-puromycin rabbit monoclonal antibody (Abclonal Technology) to detect incorporated puromycin as a measure of global translation activity.

### 2.22 Subcutaneous injections and intravenous injections

For subcutaneous tumor models, LUAD cells were resuspended in PBS ($5\times 10^{6}$ cells per 100 μL) and injected into the flanks of mice, followed by caliper measurement of tumor growth every 4 days. For intravenous metastasis models, cells ($1\times 10^{6}$ cells per 100 μL in PBS) were injected into the lateral tail vein, and tumor progression was monitored by in vivo imaging. After 4–5 weeks, mice were euthanized, and the tumors were excised, weighed, and photographed. Excised tissues were fixed and submitted to Servicebio Biotechnology (Wuhan, China) for hematoxylin and eosin (H&E) staining and immunohistochemical (IHC) analysis.

### 2.23 Statistics and reproducibility

Public transcriptomic data for lung adenocarcinoma was obtained from The Cancer Genome Atlas (TCGA, https://portal.gdc.cancer.gov) and Gene Expression Omnibus databases (GEO, https://www.ncbi.nlm.nih.gov/geo/). Differential expression analysis was performed through the DESeq2 [29] in R. All experiments were performed with at least three independent biological replicates to ensure data reliability, unless otherwise specified in the figure legends. Statistical significance was calculated using *t*-test or ANOVA based on designs of experiments. The sample size (n) indicates the number of mice for in vivo experiments and the number of independent biological replicates for cell studies. Experimental data are presented as mean ± SEM, with p < 0.05 considered statistically significant (*, p < 0.05; **, p < 0.01; ***, p < 0.001).

## 3 Results

### 3.1 tRF-5001b is present and downregulated in LUAD

We downloaded and reanalyzed the tRNA expression profiles from the GSE33858 dataset, which includes 10 paired LUAD tumor tissues and matched adjacent normal tissues. Based on the differential expression analysis, the top five most significantly dysregulated tRNAs (tRNA-Tyr-GTA, tRNA-Tyr-ATA, tRNA-Leu-TAA, tRNA-Ala-TGC, and tRNA-Lys-CTT) were selected for further screening. (Fig. 1A). In addition, we analyzed two publicly available datasets containing tRF-related expression profiles, GSE83527 [30] and GSE6218 [31], and found that tRF-5001b, which is derived from tRNA-Tyr-GTA, was significantly downregulated in both datasets (Fig. 1B). Furthermore, analysis of our previously tRNA expression profiles revealed that tRNA-Tyr-GTA was significantly decreased in LUAD tissues (Fig. 1A). Visualization of sequencing read alignment to tRNA-Tyr-GTA using Integrative Genomics Viewer (IGV) showed that the majority of reads were predominantly distributed at the 5′ end of tRNA-Tyr-GTA and exhibited high sequence similarity to tRF-5001b (Fig. S1A–C). Based on these investigations, we hypothesized that tRF-5001b may be present and may possess potential biological functions in LUAD progression.

**Fig. 1:**
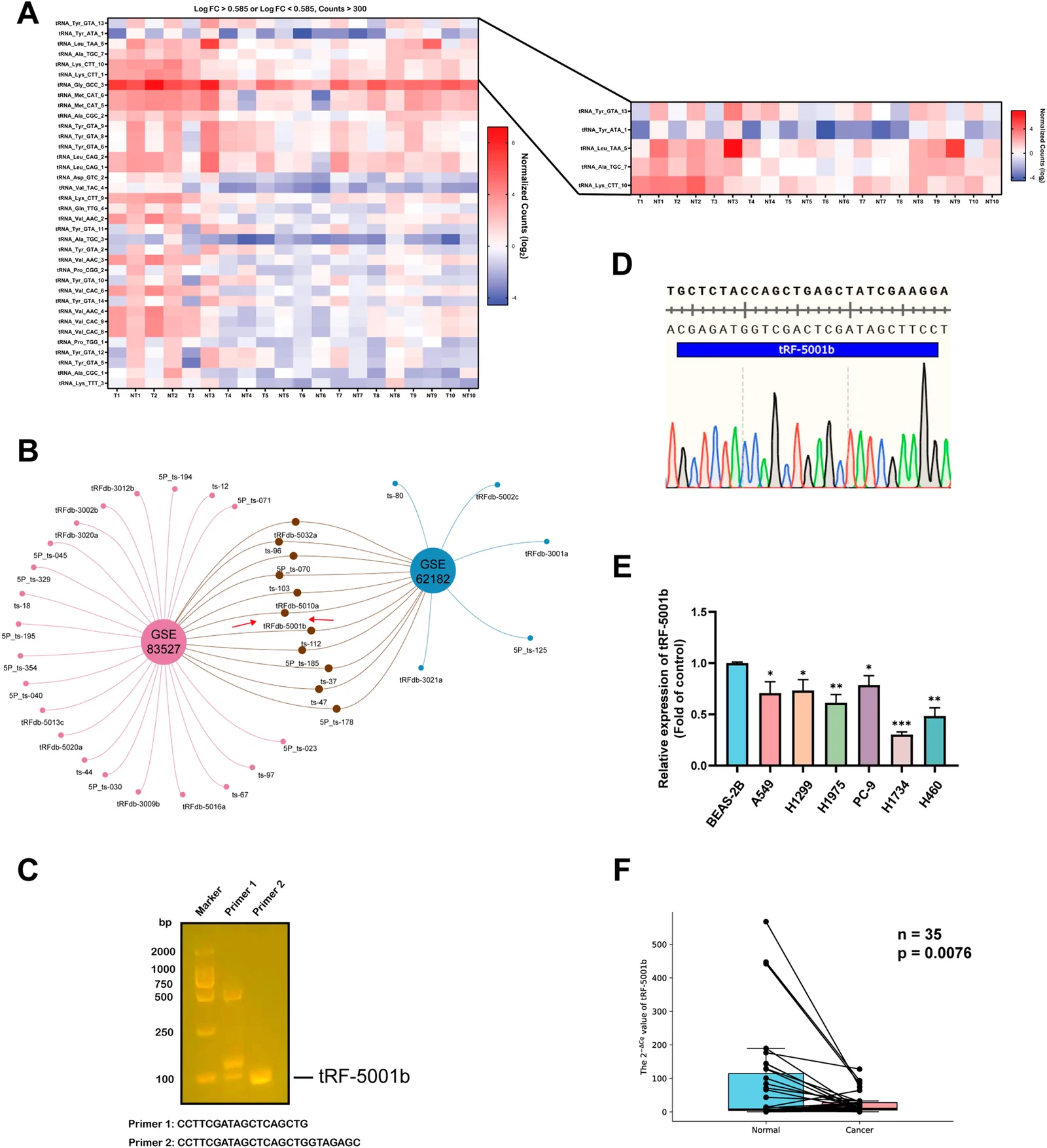
**tRF-5001b is authentically present and significantly down-regulated in lung adenocarcinoma.** (A) The hierarchical clustering heatmap illustrates down-regulated tRNA-derived fragments in cancerous and paracancerous tissues originating from 10 LUAD patients. (Log2 Fold change, Log2 FC > 0.585 and < 0.585, Counts > 300, p-value < 0.01) (B) The VEENY plot illustrates tRNA-derived fragments that are differentially expressed in LUAD from The Gene Expression Omnibus (GEO) datesets (GSE83527, GSE62182) (Log2 Fold change, Log2 FC > 0.585 and < 0.585, p-value < 0.05). (C) Agarose gel electrophoresis was used to analyze the specificity and efficiency of the amplification of tRF-5001b cDNA reverse transcribed by Add A method. (D) Sanger sequencing was used to detect fragment sequences after amplification of tRF-5001b by Add A method. (E) Expression of tRF-5001b was analyzed in LUAD cells and normal lung epithelial cells. (F) Expression of tRF-5001b was analyzed in cancerous and paracancerous tissues from 35 LUAD patients. *p < 0.05, **p < 0.01, ***p < 0.001. Three independent experiments were performed.

tRF-5001b is derived from the degradation of tRNA-Tyr-GTA and retains the 5′ end of the tRNA at a length of 25 nt. Conventional PCR is a common method for detecting tRF. However, due to the specificity of the structure, reverse transcription of tRF is commonly performed by the Add A and stem-loop methods [17], [32]. In order to explore the appropriate protocol for detecting tRF-5001b, we designed reverse transcription primers for tRF-5001b based on the introduction of tRFdb and tried to obtain the complete cDNA fragment of tRF-5001b by stem-loop method. However, the desired fragment was not acquired. Therefore, the stem-loop method is not applicable to the detection of tRF-5001b (Fig. S1D and E). Subsequently, we tried the Add A method and attempted to distinguish tRF-5001b from tRNAs. Based on the sequence of tRF-5001b and the potential break site of tRNA-Tyr-GTA, two possible PCR primers were designed. The results showed that the primers covering the full sequence of tRF-5001b could specifically amplify the target fragment of conforming size, and the accuracy of sequence was demonstrated (Fig. 1C and D).

To further demonstrate the downregulation of tRF-5001b in LUAD, we analyzed a variety of common LUAD cell lines. Consistent with the database analysis, tRF-5001b was downregulated to varying degrees in all LUAD cell lines compared to normal lung epithelial cells BEAS-2B (Fig. 1E). Clinical samples of LUAD from 35 lung cancer patients were collected and analyzed, and the results showed that the expression of tRF-5001b was significantly downregulated in LUAD tissues compared to that in paracancerous tissues (Fig. 1F). These results demonstrated that tRF-5001b is present in LUAD and that expression is generally downregulated.

### 3.2 tRF-5001b is localized in the cytoplasm and inhibits the progression of LUAD in vitro

To explore the cellular sublocalization of tRF-5001b, we verified that tRF-5001b was mainly localized to the cytoplasm of LUAD by FISH and nucleoplasmic separation experiments (Fig. 2A–C). To further investigate the effect of tRF-5001b on LUAD progression in vitro, cell lines overexpressing tRF-5001b were constructed. To specifically detect tRF-5001b and avoid potential signal interference from tRF-5001a and tRNA-Tyr-GTA, we performed Northern blot analysis using a tRF-5001b-specific probe. The results showed that only tRF-5001b was significantly overexpressed (Fig. 2D and E). CCK-8 and EdU-594 assays revealed that overexpression of tRF-5001b significantly suppressed LUAD cell proliferation, whereas inhibition of tRF-5001b enhanced cell proliferation (Fig. S2A; Fig. 2F and G). Similarly, wound healing and Transwell assays demonstrated that tRF-5001b inhibited LUAD cell migration and invasion, while tRF-5001b inhibition promoted these malignant behaviors (Fig. S2B and C; Fig. 2H and I). These results indicated that tRF-5001b inhibited the progression of LUAD in vitro.

**Fig. 2:**
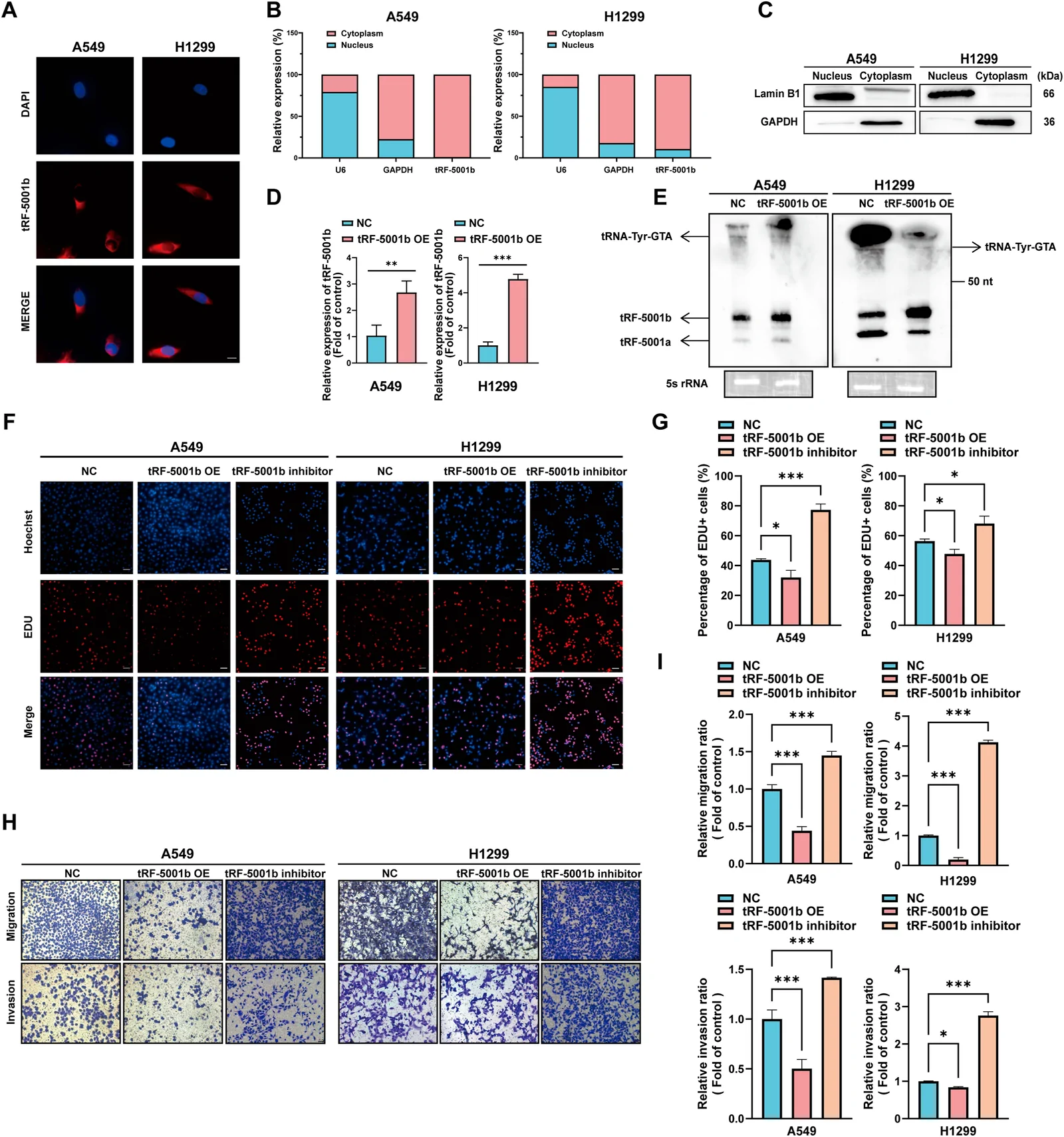
**tRF-5001b localizes to the cytoplasm in LUAD cells and inhibits LUAD proliferation and metastasis in vitro.** (A) Subcellular localization of tRF-5001b in A549 and H1299 was detected by FISH assay. Scale bar = 10 μm. (B) After cellular fractionation, the abundance of tRF-5001b in the cytoplasm and nucleus of A549 and H1299 was detected by qRT-PCR. GAPDH was used as purity control for cytoplasmic fractions. U6 snRNA was used as purity controls for nuclear fractions. (C) The quality of cytoplasmic and nuclear fractions after cellular fractionation was detected by Western blot. (D) The overexpression efficiency of tRF-5001b in A549 and H1299 was detected by qRT-PCR. (E) The overexpression efficiency of tRF-5001b in A549 and H1299 was detected by Northern blot and shows the fragment sizes of tRNA-Tyr-GTA and tRF-5001a. (F, G) EdU-594 cell proliferation assay was performed to detect the cell proliferation ability of A549 and H1299 with tRF-5001b overexpression or inhibitor. Scale bar = 50 μm. (H, I) Transwell assay was performed to detect the cell metastatic ability of A549 and H1299 with tRF-5001b overexpression or inhibitor. Scale bar = 100 μm *p < 0.05, **p < 0.01, ***p < 0.001. Three independent experiments were performed.

### 3.3 tRF-5001b intercalates with the anticodon binding domain of QARS1 in the cytoplasm

To reveal the regulation of the molecular mechanism of inhibition in LUAD by tRF-5001b, we explored the potential proteins that would interact with it. Since the sequences of tRF-5001b and tRNA-Tyr-GTA are highly repetitive, RNA-pull down should avoid interference. Thus, two different methods were designed separately and the binding protein of tRF-5001b was analyzed by mass spectrometry (Fig. 3A). To alleviate unspecific signals, a probe harboring biotin was designed to pull down the ribonucleic acid protein formed by tRF-5001b and the binding protein. At the same time, an RNA fragment with the exact same sequence as tRF-5001b was transcribed and labeled with biotin in order to bind streptavidin magnetic beads and pull down the protein molecules bound to tRF-5001b. Through protein separation and silver staining, a richer variety of proteins were revealed as the binding proteins of tRF-5001b (Fig. 3B), and mass spectrometry of the total proteins was performed to ensure that any potential binding proteins were not overlooked. Previous studies indicated that tRF functions as a competitive barrier to tRNA binding by aminoacyl-tRNA synthetases thereby inhibiting protein translation efficiency [14]. The potential binding proteins of tRF-5001b included QARS1 and Asparaginyl-tRNA synthetase 1 (NARS1). Therefore, we hypothesized that tRF-5001b may have a similar molecular mechanism (Fig. 3C).

**Fig. 3:**
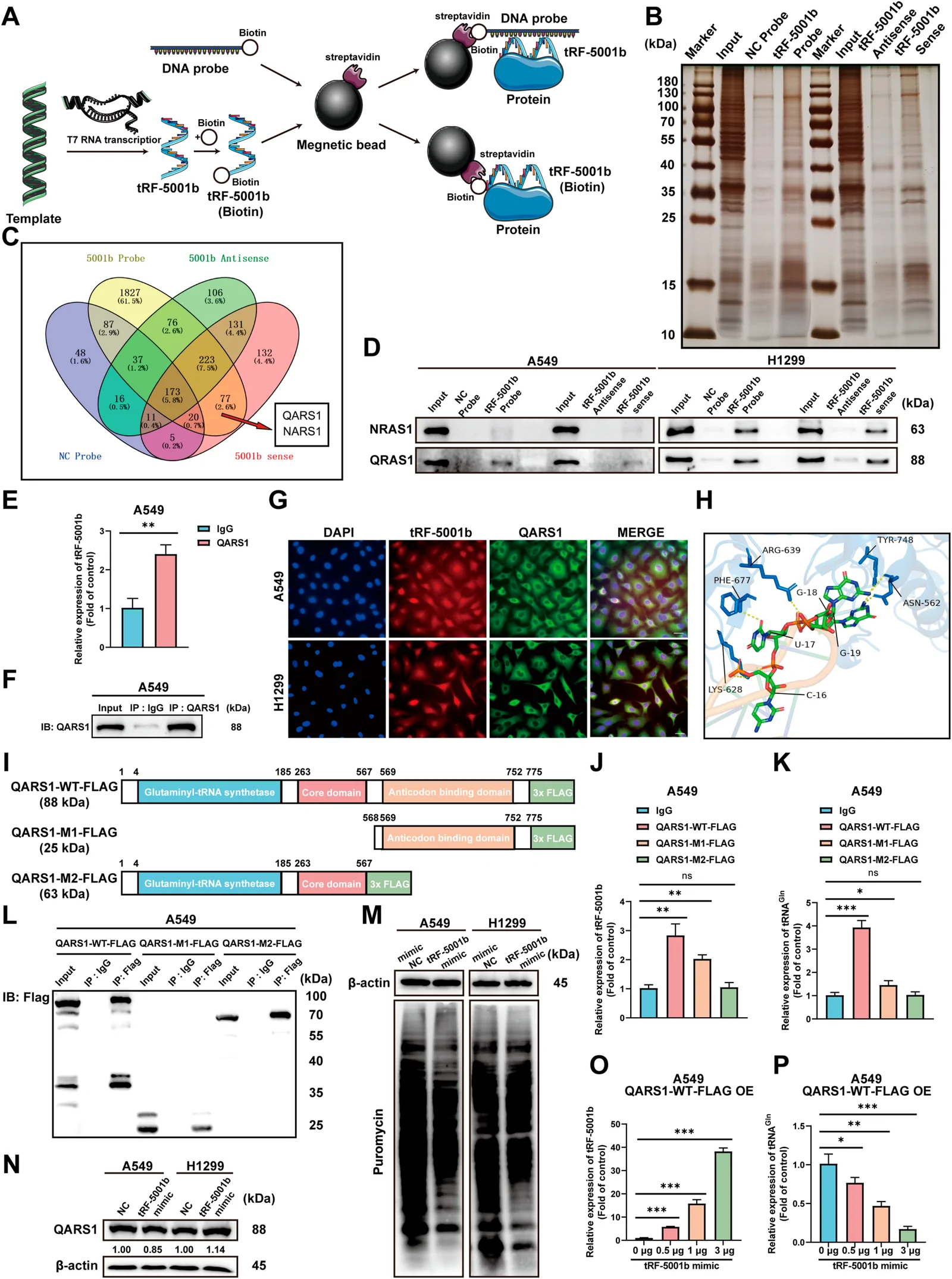
**tRF-5001b interacts with the anticodon binding domain of QARS1 and competitively prevents QARS1 from binding tRNA^Gln^.** (A) Schematic illustration of potential tRF-5001b-associated proteins enriched by RNA-pulldown assay and analyzed by mass spectrometry. (B) After SDS-PAGE separation, silver staining showed tRF-5001b-associated proteins. (C) VEENY plot indicated potential tRF-5001b-associated proteins analyzed by mass spectrometry. There are a total of 76 proteins, including QARS1 and NARS1. (D) After RNA-pulldown enrichment of tRF-5001b-associated proteins, tRF-5001b was demonstrated to bind to QARS1 by Western blot. (E) Enrichment abundance of tRF-5001b by QARS1 or IgG primary antibody was detected by RIP assay. (F) Western blot detects QARS1 protein immunoprecipitated by QARS1 or IgG primary antibody in RIP assay. (G) Subcellular co-localization of tRF-5001b and QARS1 in A549 and H1299 was detected by IF and FISH experiments. Scale bar = 20 μm. (H) Schematic illustration of the tRF-5001b and QARS1 interaction site predicted by Alphafold 3. (I) Schematic illustration of the construction of wild-type and truncated mutants of QARS1 (QARS1-WT, QARS1-M1, QARS1-M2) tagged FLAG based on the structural domain. (J, K) Enrichment abundance of tRF-5001b and tRNA^Gln^ by FLAG or IgG primary antibody was detected by RIP assay after transfection of QARS1 wild-type and mutant vectors tagged FLAG in A549. (L) Western blot detects FLAG protein immunoprecipitated by FLAG or IgG primary antibody in RIP assay after transfection of QARS1 wild-type and mutant vectors tagged FLAG in A549. (M) Puromycin intake assay detected the effect of tRF-5001b on overall translation efficiency in A549 and H1299. (N) Western blot detected whether tRF-5001b regulates the expression of QARS1. (O, P) tRF-5001b competitively inhibited QARS1 binding to tRNA^Gln^. *p < 0.05, **p < 0.01, ***p < 0.001. Three independent experiments were performed.

The binding efficiency of tRF-5001b to QARS1 and NARS1 was verified by RNA-pulldown and Western blot. Compared to NARS1, tRF-5001b was found binding more commonly to QARS1 in LUAD cell lines (Fig. 3D). Therefore, QARS1 was considered as the main target for subsequent studies. RIP experiments indicated that QARS1 could significantly enrich for tRF-5001b, and the specificity of the pull-down protein was verified by Western blot (Fig. 3E and F). To verify this interaction, the intracellular distribution of tRF-5001b and QARS1 was investigated, revealing that tRF-5001b and QARS1 were co-localized in the cytoplasm (Fig. 3G). These results indicated that tRF-5001b bound to QARS1. To further explore the molecular regulatory mechanisms exerted by tRF-5001b, specific binding sites of tRF-5001b and QARS1 were predicted by Alphafold 3, and the results indicated that tRF-5001b mightbe enriched in the anticodon binding domain of QARS1 (Fig. 3H). Next, a truncation mutant of QARS1 was constructed to verify the predicted results according to the InterPro database and labeled the expression fragments with 3×Flag (Fig. 3I). Flag-RIP experiments indicated that QARS1 (full-length)-3×Flag and a mutant retaining only the anticodon binding domain (QARS1 M1-3×Flag) were significantly enriched for tRF-5001b, whereas the mutant with a deletion of the anticodon binding domain (QARS1 M2-3×Flag) did not possess significant enrichment for tRF-5001b. In addition, tRNA^Gln^ was similarly enriched in the anticodon ring-binding structural domain (Fig. 3J–L). This phenomenon suggested that tRF-5001b might be a competitor of tRNA^Gln^ involved in translation.

### 3.4 tRF-5001b impedes the translation of HMGA2 by competitively inhibiting QARS1 binding to tRNA^Gln^

To verify the conjecture that tRF-5001b hinders translation by competitively inhibiting that QARS1 binds tRNA^Gln^, puromycin-intake assay was performed to investigate the effect of tRF-5001b on translation efficiency. The results indicated that tRF-5001b significantly inhibited the overall translation of LUAD (Fig. 3M). Moreover, tRF-5001b did not affect the integral expression of QARS1 (Fig. 3N). This indicated that tRF-5001b did not regulate translation by affecting the synthesis or degradation of QARS1. To investigate whether tRF competes with tRNA^Gln^ for binding to QARS1, a competition assay was performed in LUAD cell lines overexpressed different concentrations of tRF-5001b mimic. The results indicated that tRF-5001b significantly inhibited the ability of QARS1 binding to tRNA^Gln^ in a concentration-dependent manner (Fig. 3O and P). These results provided preliminary evidence of our speculation. To investigate the mechanisms by which tRF-5001b exerts its anti-cancer effects through the inhibition of specific protein translations, QARS1 was overexpressed in the A549 tRF-5001b OE cell line (Fig. S3A). Through proteomics analysis, we identified proteins that were downregulated in A549 tRF-5001b OE cells compared to A549 NC cells, and whose expression was restored in A549 tRF-5001b OE + QARS1 OE cells, with no significant difference observed when compared to the A549 NC group. Following this analysis, 107 candidate proteins that met the requirements were identified (Fig. 4A). Given that QARS1 is a glutaminyl transferase, the glutamine abundance of these proteins was analyzed, and the top four proteins with the highest abundance were selected for further analyzing (Fig. 4B). Considering that tRF-5001b mediated its anti-cancer effect by inhibiting translation, we hypothesized that these candidate proteins might promote oncogenesis when highly expressed in tumors. To identify which protein translation is inhibited by tRF-5001b, transcriptomic data of LUAD from TCGA was accessed and combined it with GSE81089 [33] from GEO. Analyzing by DESeq2 package in R, among four proteins, only High Mobility Group AT-hook 2 (HMGA2) was significantly overexpressed both in tumor tissues of two datasets (Fig. 4C). In recent years, HMGA2 has been reported to be involved in LUAD proliferation, invasion, and angiogenesis [34], [35], [36], [37]. Therefore, we hypothesized that tRF-5001b may inhibit the translation of HMGA2.

**Fig. 4:**
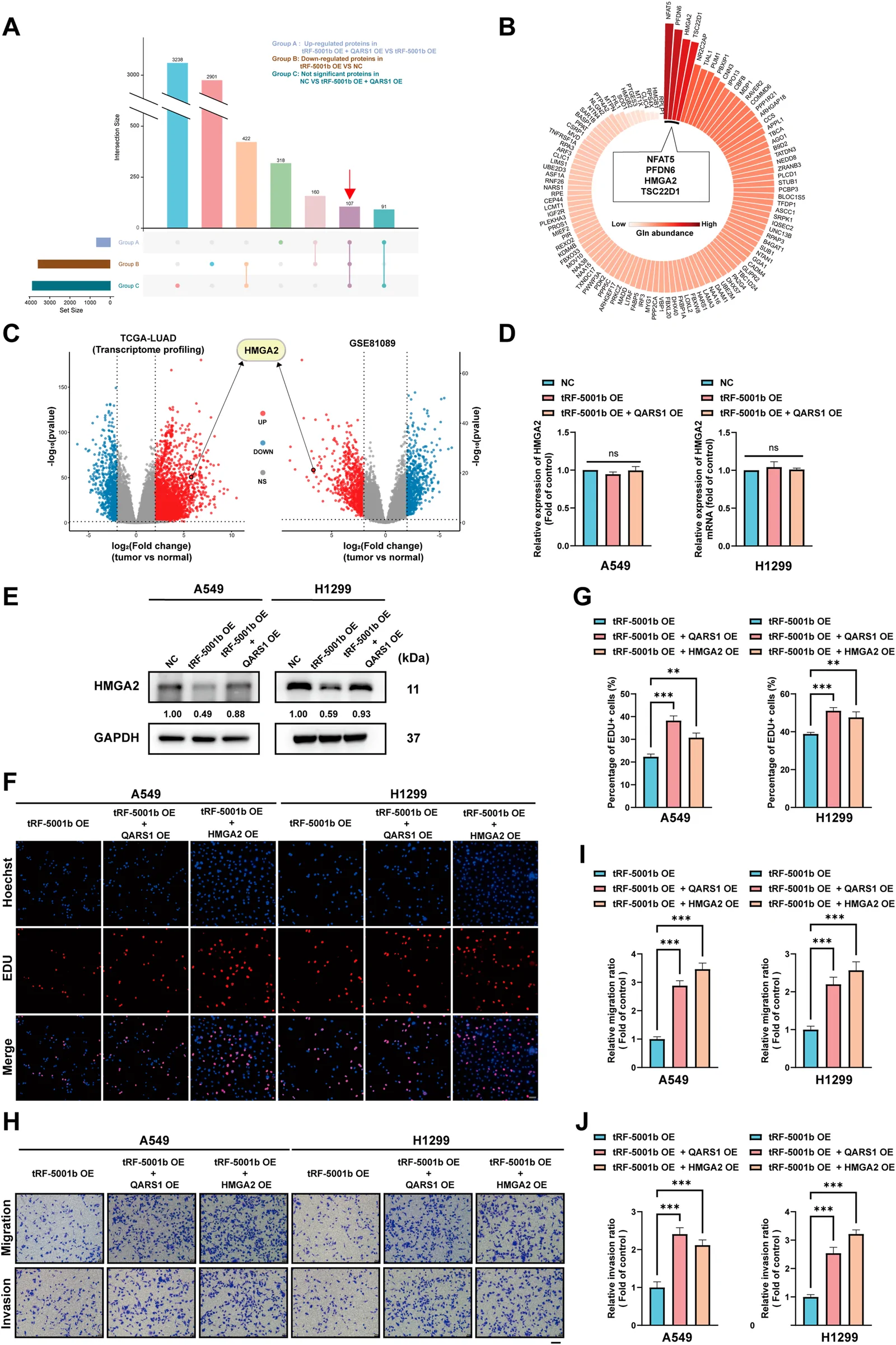
**tRF-5001b inhibits the translation of HMGA2 and tumor inhibition effects of tRF-5001b are rescued by QARS1 and HMGA2 in vitro.** (A) Proteomic analyzing found 107 potential proteins among A549 NC, A549 tRF-5001b OE and A549 tRF-5001b OE + QARS1 OE cells. (B) Gln abundance of 107 potential proteins and the top four proteins were selected to continue the following analysis. (C) Transcriptomic datasets from TCGA-LUAD and GSE81089. Both in two datasets, HMGA2 was high expression in tumor tissues contrast to normal tissues. (D) qRT-PCR detected HMGA2 expression in A549 and H1299. (E) Western blot detected HMGA2 expression in A549 and H1299. (F, G) EdU-594 cell proliferation assay was performed to detect the cell proliferation ability of A549 tRF-5001b OE and H1299 tRF-5001b OE rescued by QARS1 or HMGA2. Scale bar = 20 μm. (H-J) Transwell assay was performed to detect the cell metastatic n ability of A549 tRF-5001b OE and H1299 tRF-5001b OE rescued by QARS1 or HMGA2. Scale bar = 20 μm *p < 0.05, **p < 0.01, ***p < 0.001. Three independent experiments were performed.

To validate this hypothesis, HMGA2 mRNA levels were measured in the A549 cell lines used for proteomic sequencing as well as in H1299 cell lines, and no significant differences were observed (Fig. 4D). Western blot analysis revealed a significant downregulation of HMGA2 protein levels in A549 tRF-5001b OE cells and H1299 tRF-5001b OE cells (Fig. 4E). This suggested that HMGA2 was inhibited at the translational level. We subsequently overexpressed HMGA2 in the A549 tRF-5001b OE cell line (Fig. S3B) and performed rescue experiments. CCK-8 and EdU-594 cell proliferation assays demonstrated that both tRF-5001b OE + QARS1 OE and tRF-5001b OE + HMGA2 OE significantly reversed the inhibition of cell proliferation caused by tRF-5001b OE (Fig. S3C; Fig. 4F and G). Additionally, wound healing and Transwell assays showed that tRF-5001b OE + QARS1 OE and tRF-5001b OE + HMGA2 OE significantly reversed the inhibition of migration and invasion induced by tRF-5001b OE (Fig. S3D–E; Fig. 4H–J). These results suggested that tRF-5001b inhibited HMGA2 translation by competitively binding to QARS1with tRNA^Gln^.

### 3.5 tRF-5001b inhibits LUAD proliferation and metastasis in vivo

To investigate the antitumor function of tRF-5001b against LUAD in vivo, we subcutaneously injected A549 cells (5*10^6^) expressing tRF-5001b or NC into BALB/c nude mice to construct xenograft mouse models (Fig. 5A). Tumor volume was observed and measured every 4 days, and tumor weighing was performed 4-5 weeks after injection. The results indicated that overexpression of tRF-5001b significantly inhibited the growth of LUAD tumors in mice (Fig. 5B–E). Immunohistochemical results indicated that the proliferation marker Ki-67 and migration marker MMP-2 were significantly downregulated in tRF-5001b overexpression tumors tissues. Furthermore, HMGA2 was also inhibited by tRF-5001b in vivo, which is consistent with the results observed in the cellular functional assays (Fig. 5F). To further investigate the effect of tRF-5001b on LUAD metastasis in vivo, a lung metastasis model of LUAD was constructed by caudal vein injection of A549 tRF-5001b OE or A549 NC, both with luciferase (Fig. 5G). An in vivo imaging system (IVIS) was used to monitor tumor growth over a five-week period. The luminescence intensity of tumors was observed and the tRF-5001b OE group remained consistently lower than that in the NC group (Fig. 5H). Histopathological analysis further supported these findings. Hematoxylin and eosin (H&E) staining of lung tissues revealed extensive infiltrative tumor growth in the NC group, whereas the tRF-5001b OE group largely retained intact alveolar structures with only minimal tumor cell presence (Fig. 5I). Together, these results demonstrated that tRF-5001b suppressed tumor growth and metastasis in vivo.

**Fig. 5:**
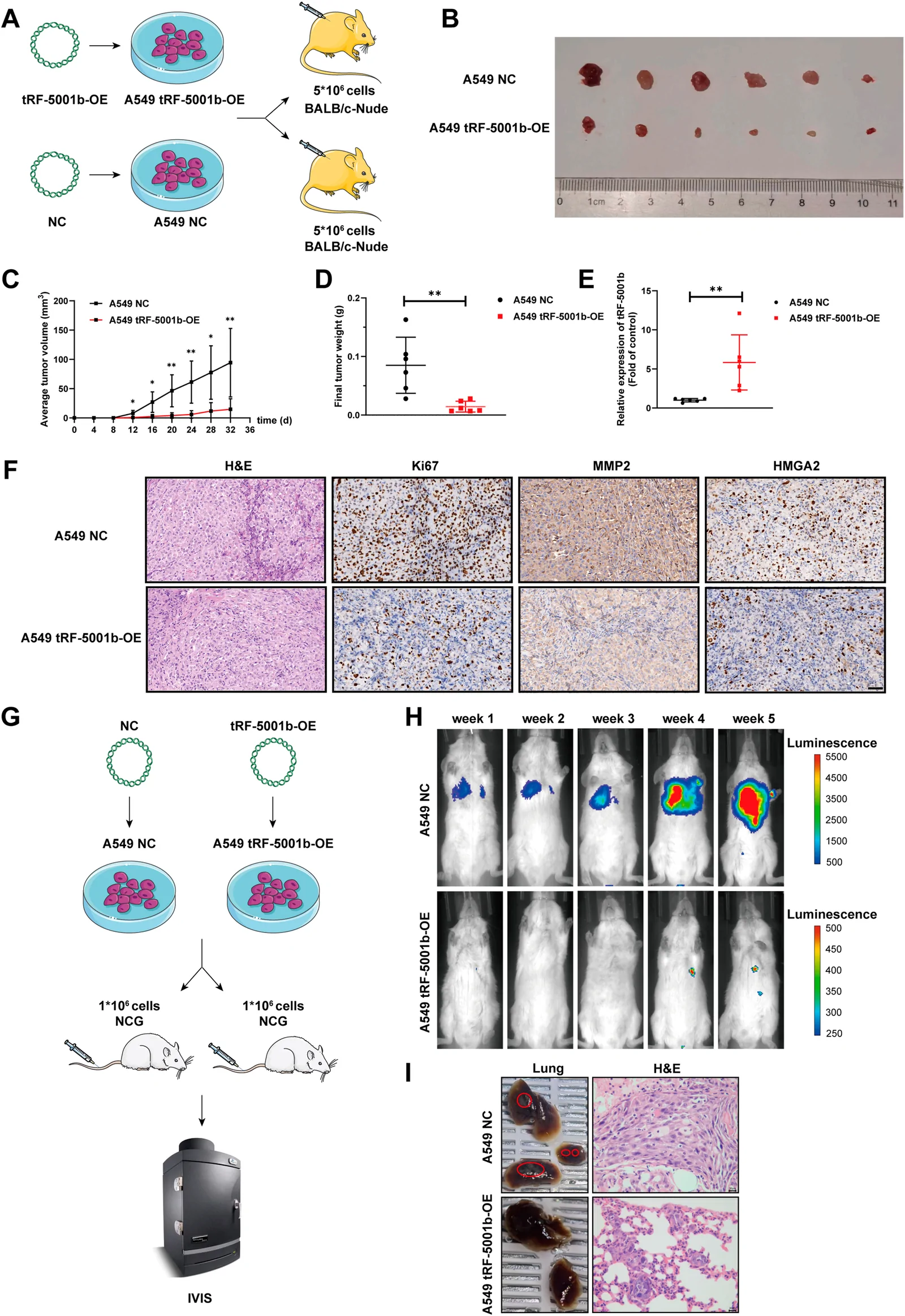
**tRF-5001b inhibits the growth and metastasis of LUAD in vivo.** (A) Schematic illustration of the construction of xenograft mouse models. (B) Morphology and size of negative control and xenograft tumors overexpressing tRF-5001b after 5 weeks of growth. (C) Statistical analysis of the volume of NC and tRF-5001b overexpressing xenograft tumors. (D) Statistical analysis of the weight of NC and tRF-5001b overexpressing xenograft tumors. (E) qRT-PCR detects the expression of tRF-5001b in NC and tRF-5001b overexpressing xenograft tumors. (F) NC and tRF-5001b overexpressing xenograft tumors were stained with hematoxylin and eosin solutions. Protein levels of Ki67, MMP2, HMGA2 were detected by IHC. Scale bar = 20 μm. (G) Caudal vein injection of NC or tRF-5001b overexpressing of A549 transfected luciferase expression vector was performed to construct a lung metastasis model of LUAD, and tumor size was detected by IVIS. (H) Luminescence detected by IVIS in NC or tRF-5001b overexpressing groups of mice. (I) Lung tissues before or after hematoxylin and eosin solution staining in NC or tRF-5001b overexpressing groups of mice. Scale bar = 20 μm *p < 0.05, **p < 0.01, ***p < 0.001. Three independent experiments were performed.

### 3.6 METTL1 catalyzes the m^7^G modification of tRNA-Tyr-GTA to inhibit the biogenesis of tRF-5001b

tRF-5001b is a 5′-tRNA-derived fragment originating from tRNA-Tyr-GTA. METTL1, together with its regulatory partner WDR4, is the major cellular methyltransferase complex responsible for m^7^G modification of a subset of tRNAs, and tRNA-Tyr-GTA has been identified among METTL1-regulated m^7^G-modified tRNAs [22], [38], [39]. Previous studies have further demonstrated that METTL1/WDR4-mediated m^7^G modification regulates tRNA stability and METTL1 is expressed in high abundance in LUAD clinical samples [20], [26], [40]. Based on the investigation, we demonstrated that both METTL1 and WDR4 were expressed upregulated in LUAD cell lines (Fig. 6A). Next, we further explored whether the downregulation of tRF-5001b was mediated by the METTL1/WDR4 methyltransferase complex. Through RIP assay, tRNA-Tyr-GTA, generating tRF-5001b mediated by ribonucleic acid endonuclease, was enriched by METTL1 and had a significant m^7^G modification (Fig. 6B–D). To further investigate whether METTL1 regulates the biogenesis of tRF-5001b by catalyzing m^7^G modification, the expression of METTL1 was knocked down in the LUAD cell line (Fig. 6E and F). The results indicated that down-regulation of METTL1 significantly reduced the stability and expression of tRNA-Tyr-GTA, which is consistent with previous studies (Fig. 6G and H). On this basis, the expression of tRF-5001b was significantly upregulated after knockdown of METTL1 by Northern blot (Fig. 6I). In addition, the expression of WDR4 was knocked down to demonstrate that tRF-5001b biogenesis is regulated by methyltransferases rather than METTL1 alone (Fig. 6J and K). These data indicated that the regulatory function of WDR4 on tRNA-Tyr-GTA and tRF-5001b was consistent with METTL1 (Fig. 6L and M). Therefore, we preliminarily revealed that METTL1 promoted the biogenesis of tRF-5001b by catalyzing the m^7^G modification of tRNA-Tyr-GTA.

**Fig. 6:**
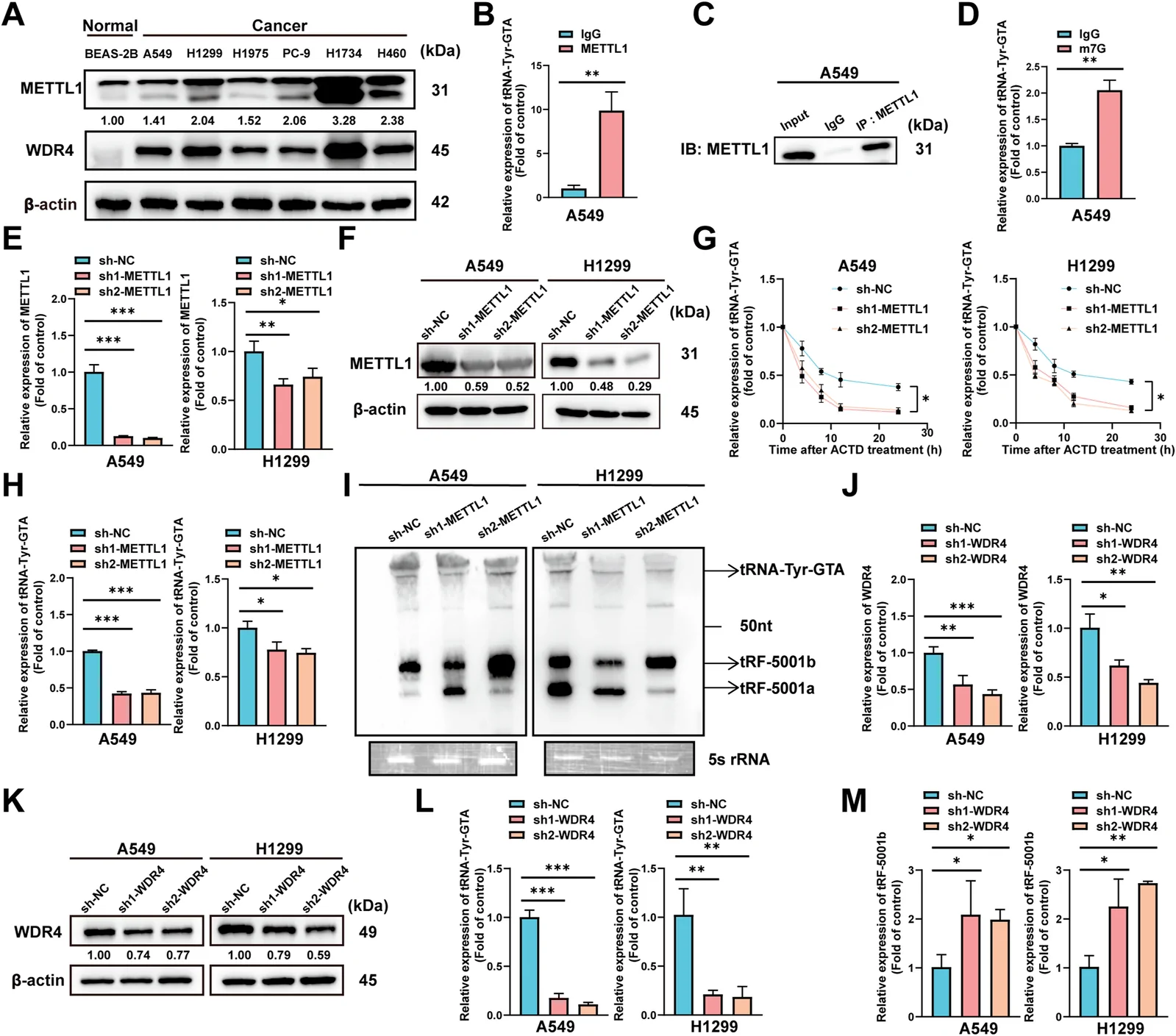
**METTL1 catalyzes the m^7^G modification of tRNA-Tyr-GTA and inhibits the biogenesis of tRF-5001b.** (A) Western blot was performed to detect the expression of METTL1 and WDR4 in LUAD cell lines. Representative images are shown. (B) Enrichment abundance of tRNA-Tyr-GTA by METTL1 or IgG primary antibody was detected by RIP assay. (C) Western blot detects METTL1 protein immunoprecipitated by METTL1 or IgG primary antibody in RIP assay. (D) Enrichment abundance of tRNA-Tyr-GTA by m^7^G or IgG primary antibody was detected by RIP assay. (E, F) The knockdown efficiency of METTL1 in A549 and H1299 was detected by qRT-PCR and Western blot. (G) Variation in RNA abundance of tRNA-Tyr-GTA after treatment with actinomycin D (2 μg/mL) in A549 and H1299 (NC and METTL1 knockdown). (H) qRT-PCR was performed to detect the expression of tRNA-Tyr-GTA in A549 and H1299 after METTL1 knockdown. (I) Northern blot was performed to detect the expression of tRF-5001b in A549 and H1299 after METTL1 knockdown and shows the fragment sizes of tRNA-Tyr-GTA and tRF-5001a. (J, K) The knockdown efficiency of WDR4 in A549 and H1299 was detected by qRT-PCR and Western blot. (L) qRT-PCR was performed to detect the expression of tRNA-Tyr-GTA in A549 and H1299 after WDR4 knockdown. (M) qRT-PCR was performed to detect the expression of tRF-5001b in A549 and H1299 after WDR4 knockdown. *p < 0.05, **p < 0.01, ***p < 0.001. Three independent experiments were performed.

To further demonstrate that METTL1 inhibited the biogenesis of tRF-5001b by catalyzing the m^7^G modification of tRNA-Tyr-GTA and explored the role of this mechanism in the development of LUAD, A549 cell lines with wild-type METTL1-Flag (METTL1-WT-Flag) or catalytic dead mutant METTL1-Flag (METTL1-MUT-Flag) were established to investigate whether the function performed by the methylase activity is necessary (Fig. 7A). We performed Flag-RIP (Fig. 7B) and m^7^G-RIP (Fig. 7C) and observed that METTL1-WT-FLAG was significantly enriched for tRNA-Tyr-GTA and promoted more m^7^G modifications while METTL1-MUT-Flag lost this function (Fig. 7B and C). Subsequently, METTL1-WT and METTL1-MUT were overexpressed in LUAD cell lines separately (Fig. 7D). METTL1-WT significantly contributed to the stability and expression efficiency of tRNA-Tyr-GTA and the downregulation of tRF-5001b mediated by METTL1-WT was rescued by 5001b mimic. However, METTL1-MUT lost its function to regulate tRNA-Tyr-GTA and tRF-5001b (Fig. 7E and F). On the basis of rescue experiments, the effect of METTL1 was further explored on the development of LUAD by regulating the biogenesis of tRF-5001b. The results indicated that METTL1-WT significantly promoted proliferation, migration and invasion of LUAD in vitro. However, METTL1-MUT was unable to exert normal regulatory functions (Fig. 7G–J). Taking together, METTL1 inhibited the biogenesis of tRF-5001b by catalyzing the m^7^G modification of tRNA-Tyr-GTA to regulate LUAD. This mechanism explained the downregulation of tRF-5001b in LUAD.

**Fig. 7:**
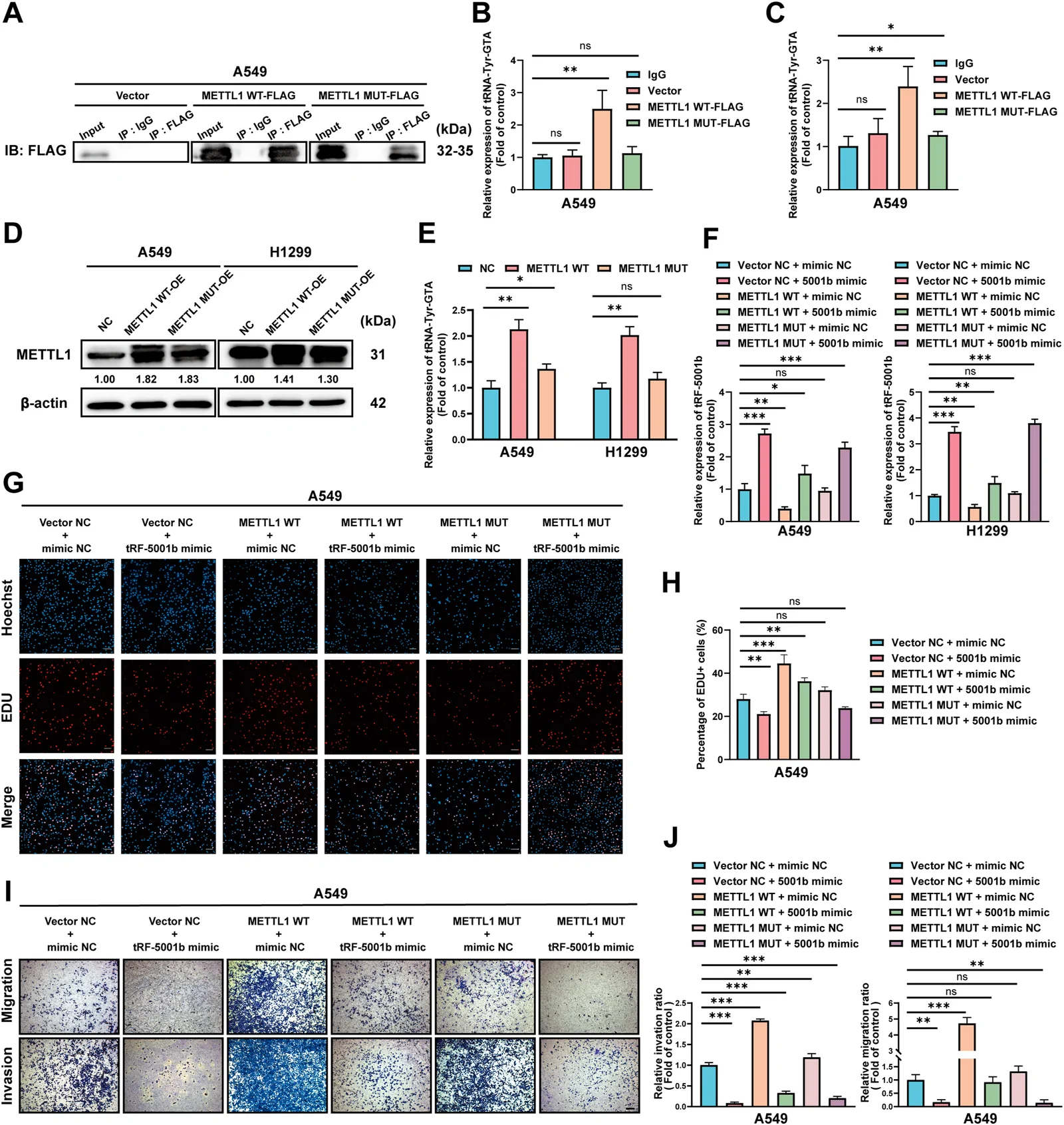
**Methylase activity of METTL1 is essential for the regulation of the biogenesis of tRF-5001b and LUAD development.** (A) Western blot was performed to detect FLAG protein immunoprecipitated by FLAG or IgG primary antibody in RIP assay after transfection of pCDH, wild type and catalytic dead mutant METTL1 vectors tagged FLAG in A549. (B) Enrichment abundance of tRNA-Tyr-GTA by FLAG or IgG primary antibody was detected by RIP assay after transfection of vector, wild type and catalytic dead mutant METTL1 vectors tagged FLAG in A549. (C) Enrichment abundance of tRNA-Tyr-GTA by m7G or IgG primary antibody was detected by RIP assay after transfection of vector, wild type and catalytic dead mutant METTL1 vectors tagged FLAG in A549. (D) Western blot was performed to detect the overexpression efficiency of NC, wild type and catalytic dead mutant METTL1 in A549 and H1299. (E) qRT-PCR was performed to detect the expression of tRNA-Tyr-GTA after overexpression of NC, wild type and catalytic dead mutant METTL1 in A549 and H1299. (F) qRT-PCR was performed to detect the expression of tRF-5001b in wild-type or catalytic dead mutant METTL1 and tRF-5001b mimic or mimic NC in A549 and H1299. (G, H) EdU-594 cell proliferation assay was performed to detect the cell proliferation ability of A549 with wild-type METTL1 and tRF-5001b overexpression. Scale bar = 20 μm. (I, J) Transwell assay was performed to detect the cell metastasis and migration ability of A549 with wild-type METTL1 and tRF-5001b overexpression. Scale bar = 20 μm *p < 0.05, **p < 0.01, ***p < 0.001. Three independent experiments were performed.

## 4 Discussion

tRFs were considered to be degraded fragments after tRNAs metabolism in the past, with little recognition of their biological functions [41]. However, recent studies have unveiled several tRFs that play significant roles in the biological processes of LUAD, primarily by influencing transcriptional regulation to control LUAD development [10], [42], [43], [44]. In this study, we present evidence for the realistic presence of tRF-5001b in LUAD, marking it as the first tRF shown to act at the translational level to inhibit LUAD progression.

Lung cancer continues to be the leading cause of cancer-related mortality worldwide [3]. Our analysis of transcriptomic data revealed that tRF-5001b was significantly downregulated in cancer tissues of LUAD patients. Further in vivo and in vitro functional assays demonstrated that tRF-5001b inhibited LUAD proliferation and invasion. Therefore, tRF-5001b has the potential to be developed as a small-molecule drug to counteract the progression of LUAD.

Understanding the biogenesis of tRFs is crucial for uncovering their functional roles. Nucleic acid endonucleases, such as Dicer, Angiogenin (ANG), and RNase2, are essential for tRF generation, with early studies suggesting that tRNA modifications can influence the efficiency of their biogenesis [45]. Recently, METTL1/WDR4-mediated m^7^G tRNA modification has been shown to stabilize tRNAs by protecting them from degradation [26]. We discovered that a similar mechanism also exists in the biogenesis of tRF-5001b. Specifically, the m^7^G modification mediated by METTL1/WDR4 enhanced the stability of tRNA-Tyr-GTA and inhibited the biogenesis of tRF-5001b. However, other mechanisms might also contribute to the regulation of tRF-5001b biogenesis. Notably, METTL1/WDR4 catalyzes m^7^G46 modification on specific full-length tRNAs, whereas tRF-5001b is a 5′-tRF which does not contain the canonical G46 modification site [22], [24]. Moreover, tRNA^Gln^ has been experimentally shown to be a non-m^7^G-modified tRNA and is not substantially affected by METTL1/WDR4 perturbation [39], [40]. Therefore, there is currently no direct evidence that METTL1/WDR4 modifies tRF-5001b itself or tRNA^Gln^.

The functional potential of tRFs appears to be linked to their ability to retain part of the complex structure of their parent tRNAs. This unique characteristic enables them to carry out a variety of biological functions, distinguishing them from other prevalent small RNAs [2], [46]. In our study, we found that tRF-5001b, due to its sequence similarity to tRNA, is capable of occupying the binding site of tRNA^Gln^ on QARS1. This interaction prevented QARS1 from binding to tRNA^Gln^, thereby hindering translation progression.

Through proteomic analysis, we identified HMGA2, a well-established chromatin-associated oncogenic factor, as a potential downstream target of tRF-5001b and hypothesized that tRF-5001b might suppress its translation. HMGA2 is known to play a critical role in cancer progression and metastasis and is frequently upregulated in LUAD, where its elevated expression has been associated with poor prognosis [47]. Functional studies have further demonstrated that HMGA2 promotes lung cancer cell proliferation, migration, invasion, and epithelial–mesenchymal transition (EMT), partly through the regulation of EMT-related factors such as Twist 1 [48], [49], [50]. Similar pro-metastatic functions of HMGA2 have also been reported in other malignancies, supporting its broader importance in tumor progression [51], [52], [53]. Consistent with these previous findings, our rescue experiments demonstrated that overexpression of either QARS1 or HMGA2 could reverse the inhibitory effects of tRF-5001b on LUAD cell proliferation and invasion, further supporting the proposed mechanistic framework. However, it should be noted that direct evidence at the ribosomal level was still lacking, as we were unable to definitively demonstrate a reduction in the translational efficiency of HMGA2.

As the field continues to evolve, recent advancements in understanding the RNA histology and proteomic features of LUAD, as well as the analysis of specific signaling pathways, have contributed significantly to improving clinical diagnostics for LUAD [54], [55], [56]. However, challenges remain in developing more reliable diagnostic protocols due to the absence of early symptoms in LUAD [57], [58], [59]. Given this, further exploration into effective diagnostic markers and therapeutic targets is crucial. Considering the low expression of tRF-5001b in tumors and its potential tumor-suppressive effects, it presents an exciting opportunity to become a new diagnostic marker and therapeutic target for LUAD.

## 5 Conclusion

Our findings suggest that tRF-5001b, regulated by METTL1/WDR4, plays a critical role in inhibiting LUAD progression by competitively hindering the binding of QARS1 to tRNA^Gln^, thus suppressing HMGA2 translation (Fig. 8). This discovery is the first time a tRF has been shown to exert its tumor-suppressive effects at the translation level in LUAD. Our study provides novel mechanistic insights into the antitumor efficacy of tRFs, offering new strategies for the early diagnosis and treatment of LUAD.

**Fig. 8:**
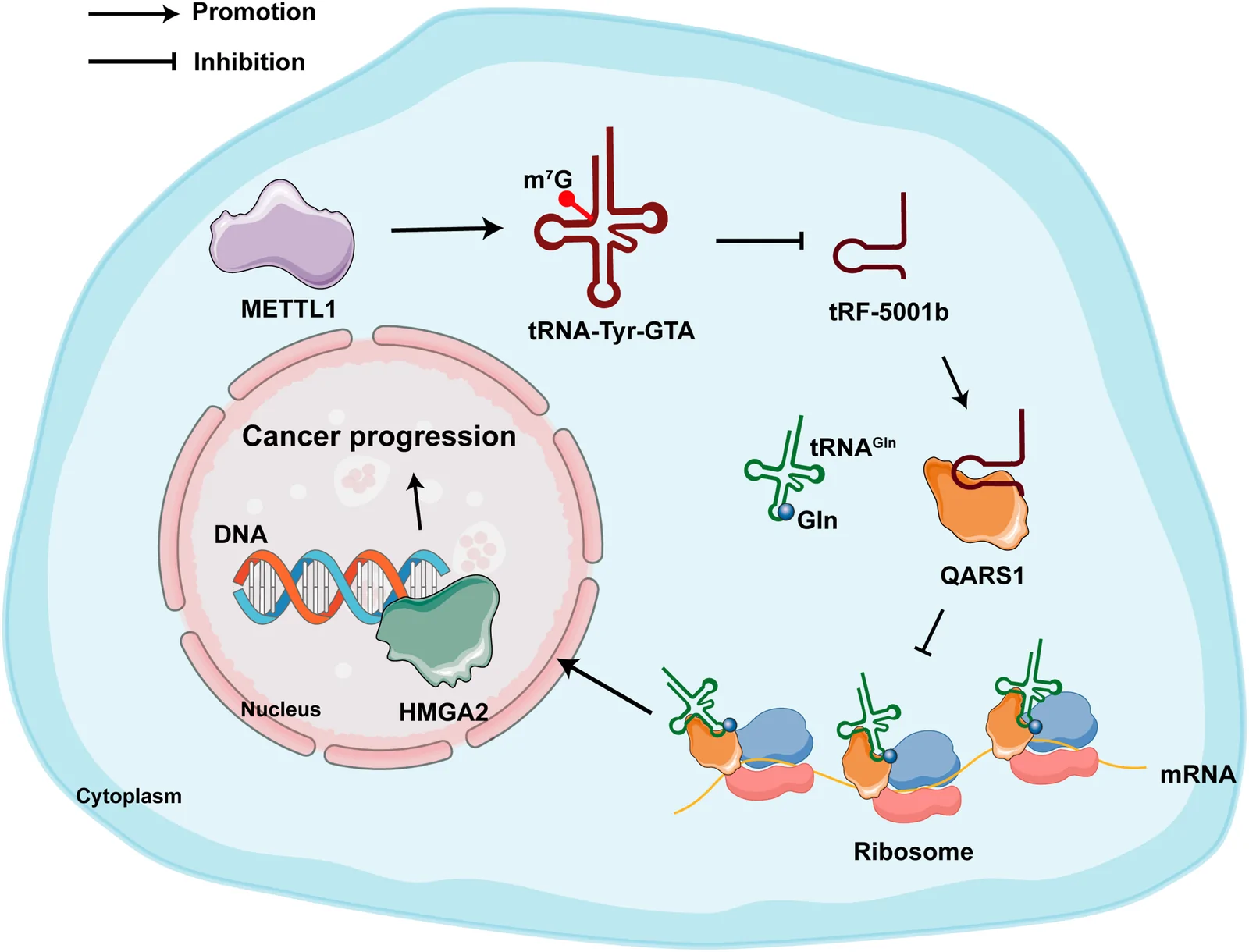
A model illustrating that tRF-5001b suppresses LUAD progression by competitively disrupting QARS1–tRNA^Gln^ interaction and inhibiting HMGA2 translation, while METTL1 regulates tRF-5001b biogenesis through m^7^G modification of tRNA-Tyr-GTA.

## CRediT authorship contribution statement

**Jingyuan Wang:** Writing – original draft. **Fan Wu:** Writing – original draft. **Yanli Li:** Writing – review & editing. **Zhiyu Wu:** Investigation. **Qiyue Song:** Formal analysis, Conceptualization. **Weiwei Wang:** Writing – review & editing. **Qiangling Sun:** Writing – review & editing. **Zhongliang Ma:** Writing – review & editing.

## Ethics declaration

Written informed consent to take part in the study and to publish the article has been obtained from all participants or their legal representatives. The privacy rights of participants have been observed.

This study included organ or tissue donors. This study includes human biological material and consent was obtained by donors, or their next of kin or legal representatives, for use in this study and for publication of the article. The samples used in this research were not sourced from executed prisoners or prisoners of conscience.

This study was performed in compliance with relevant laws, regulatory frameworks and guidelines where the research took place. This study was approved by the Ethics Committee of the Yunnan Cancer Hospital. (Approval No. SLKYLX 2025-317)

This study was conducted in accordance with the ARRIVE (Animal Research: Reporting of In Vivo Experiments) guidelines. This study was approved by the Ethics Committee of Shanghai University. (Approval No. YS 2025-030)

## Data availability

The data that support the findings of this study are available from the corresponding author upon reasonable request.

## Funding

This study received no funding support.

## Declaration of competing interest

The authors declare that they have no known competing financial interests or personal relationships that could have appeared to influence the work reported in this paper.

## List of abbreviations

LUAD, lung adenocarcinoma; tRFs, tRNA-derived fragments; EdU, ethynyl deoxyuridine; CCK-8, Cell Counting Kit-8; QARS1, glutaminyl-tRNA synthetase 1; HMGA2, high mobility group AT-hook 2; qRT-PCR, quantitative reverse transcription PCR; METTL1, methyltransferase like 1; WDR4, WD repeat-containing protein 4; nt, nucleotides; TCEA3, transcription elongation factor A3; MED29, mediator complex subunit 29; HSPB1, heat shock protein family B member 1; LARS2, leucine-tRNA synthetase 2; METTL, methyltransferase-like; SAM, S-adenosyl methionine; m7G, N7-methylguanosine; ATCC, American Type Culture Collection; HEK-293T, human embryonic kidney 293T; BEAS-2B, human bronchial epithelial cells; DMEM, Dulbecco's modified Eagle's medium; RPMI-1640, Roswell Park Memorial Institute-1640; SPF, specific pathogen–free; DH5α, DH5alpha competent cells; GAPDH, glyceraldehyde-3-phosphate dehydrogenase; *E. coli*, *Escherichia coli*; RIPA, radioimmunoprecipitation assay; PMSF, phenylmethylsulfonyl fluoride; PVDF, polyvinylidene difluoride; TBST, Tris-buffered saline with Tween 20; HRP, horseradish peroxidase; ECL, enhanced chemiluminescence; SDS-PAGE, sodium dodecyl sulfate-polyacrylamide gel electrophoresis; TBE, Tris-borate-EDTA; FISH, fluorescence in situ hybridization; DAPI, 4′,6-diamidino-2-phenylindole; IP, immunoprecipitation; RIP, RNA immunoprecipitation; TCGA, The Cancer Genome Atlas; GEO, Gene Expression Omnibus; ANOVA, analysis of variance; SEM, standard error of the mean; NARS1, asparaginyl-tRNA synthetase 1; OE, overexpression; mRNA, messenger RNA; NC, negative control; BALB/c, BALB/c mice; Ki-67, marker of proliferation Ki-67; MMP-2, matrix metalloproteinase-2; IVIS, in vivo imaging system; H&E, hematoxylin and eosin; Dicer, Dicer endoribonuclease; ANG, angiogenin; RNase2, ribonuclease A family member 2; EMT, epithelial–mesenchymal transition; IGV, Integrative Genomics Viewer.
