# Supplementary material for: METTL1-regulated tRF-5001b suppresses lung adenocarcinoma by impairing QARS1 interaction with tRNAGln and inhibiting HMGA2 translation
Source: Noncoding RNA Res. 2026 Nov 3;21:74–90. doi: 10.1016/j.ncrna.2026.09.003 (PMC13756536; doi:10.1016/j.ncrna.2026.09.003)
Supplement: Supplementary file 1 [file mmc1.pdf]

Supplementary Material 1.
Figure S1-S3 and Supplementary Table 1

METTL1-regulated tRF-5001b suppresses lung adenocarcinoma by impairing QARS1 interaction with $tRNA^{Gln}$ and inhibiting HMGA2 translation

Jingyuan Wang, Fan Wu, Yanli Li, Zhiyu Wu, Qiyue Song, Weiwei Wang, Qiangling Sun, Zhongliang Ma

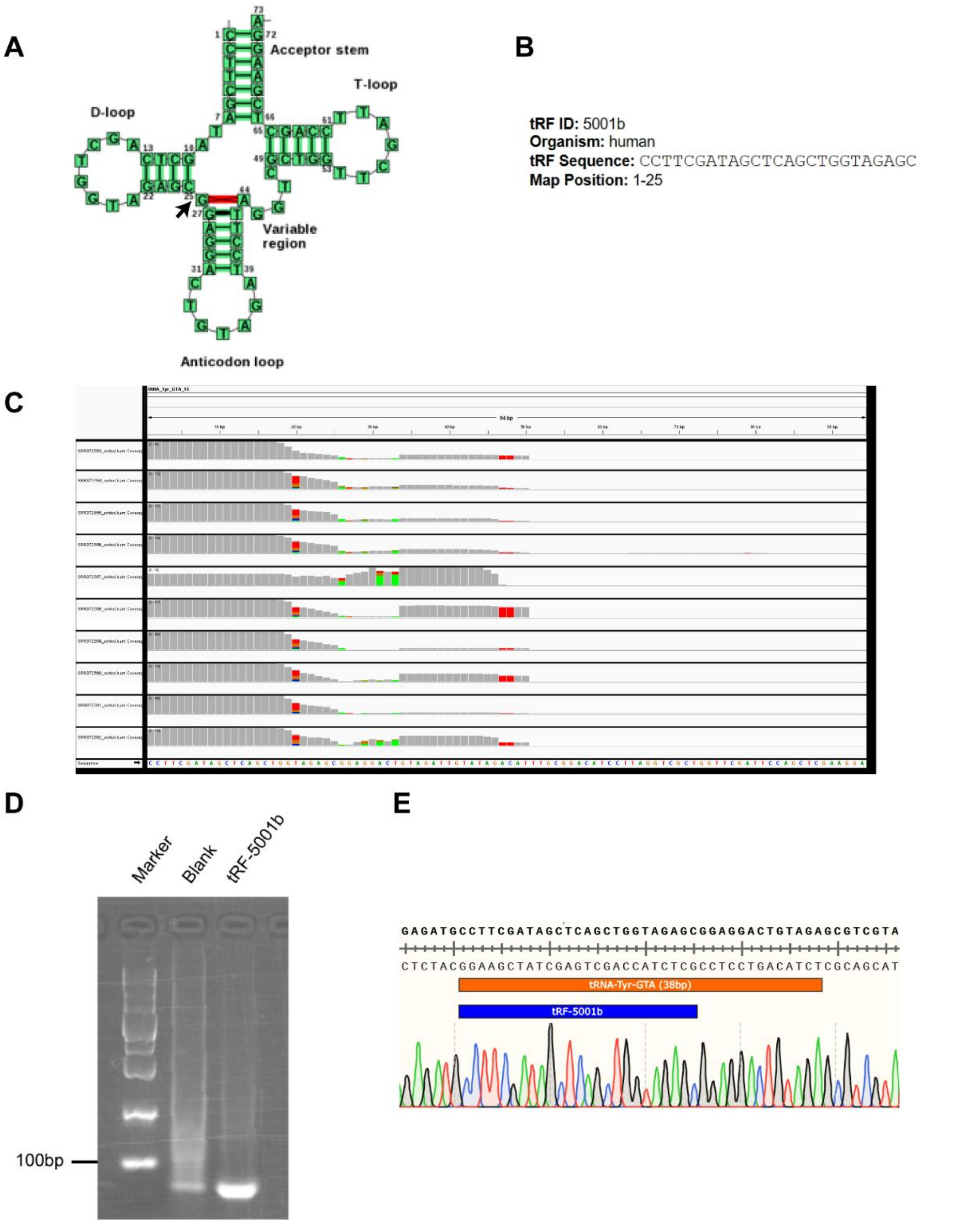

Figure S1. Identification of the realistic presence of tRF-5001b in LUAD. (A) The origin of tRF-5001b and the cleavage site on tRNA-Tyr-GTA. (B) Introduction of tRF-5001b in the tRFdb database. (C) Results of comparative sequence analysis of all small RNA from small RNA expression profiles with tRNA-Tyr-GTA. (D) Agarose gel electrophoresis was used to analyze the specificity and efficiency of the amplification of tRF-5001b cDNA reverse transcribed by stem-loop method. (E) Sanger sequencing was used to detect fragment sequences after amplification of tRF-5001b by stem-loop method.

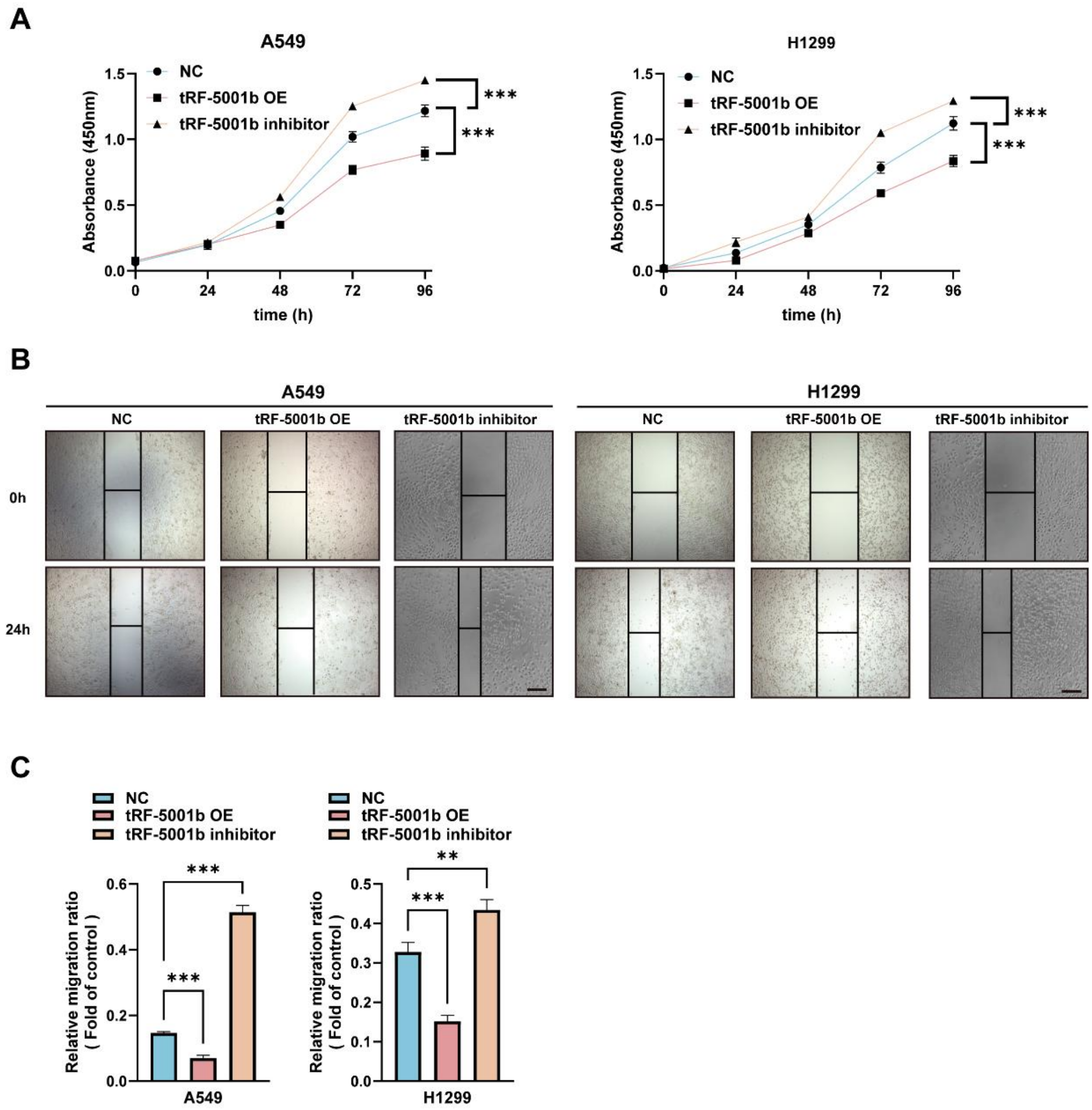

Figure S2. tRF-5001b inhibits LUAD proliferation and migration in vitro. (A) CCK-8 assay was performed to detect the cell proliferation ability of A549 and H1299 with tRF-5001b overexpression or inhibitor. (B, C) Would healing was performed to detect the cell migration ability of A549 and H1299 with tRF-5001b overexpression or inhibitor. Scale bar = 100 μm. *p < 0.05, **p < 0.01, ***p < 0.001. Three independent experiments were performed.

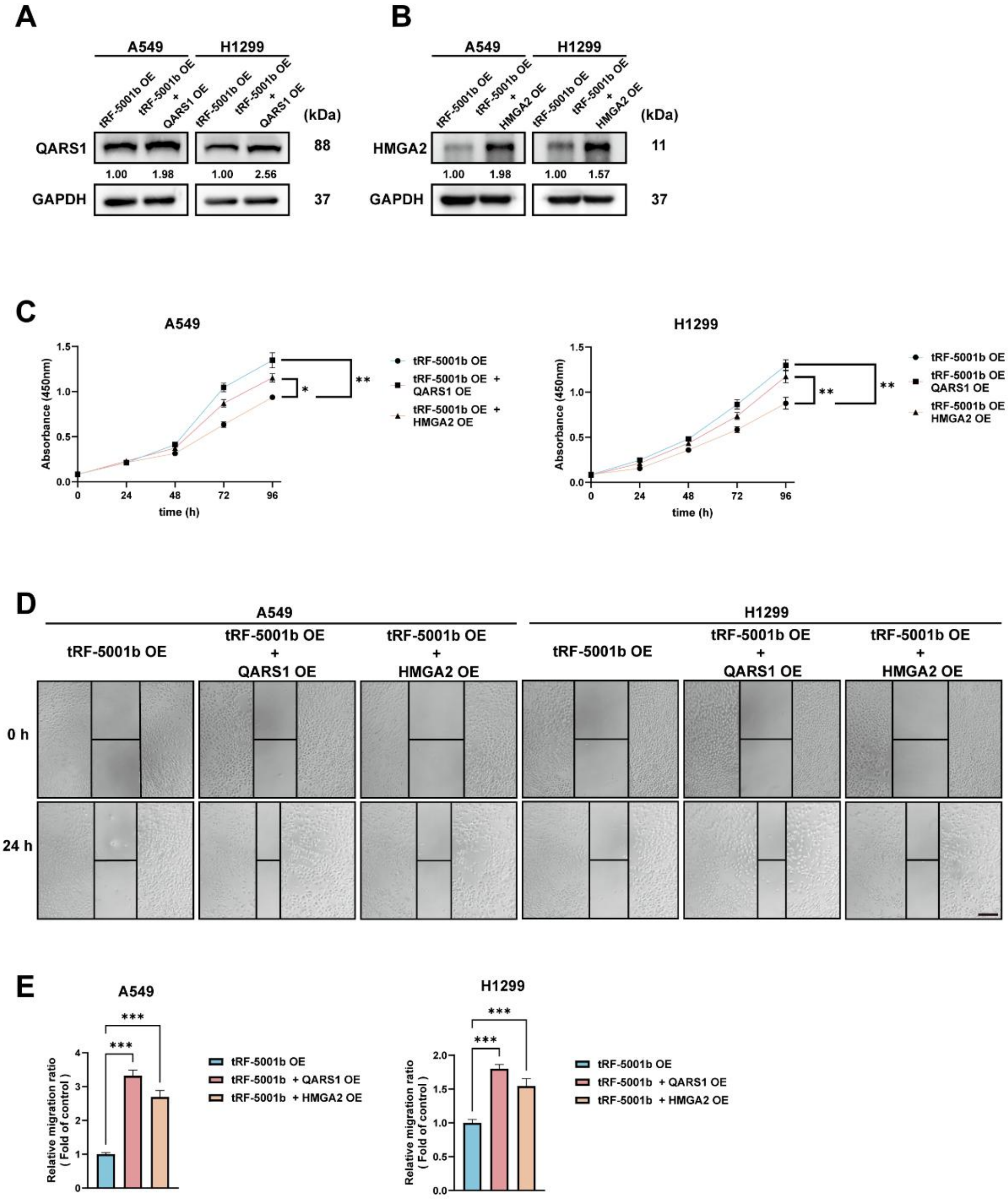

Figure S3. Tumor inhibition effects of tRF-5001b are rescued by QARS1 and HMGA2 in vitro. (A) Western blot detected QARS1 overexpressed in A549 and H1299. (B) Western blot detected HMGA2 overexpressed in A549 and H1299. (C) CCK-8 assay was performed to detect the cell proliferation ability of A549 tRF-5001b OE and H1299 tRF-5001b OE rescued by QARS1 or HMGA2. (D, E) Would healing was performed to detect the cell migration ability of A549 tRF-5001b OE and H1299 tRF-5001b OE rescued by QARS1 or HMGA2. Scale bar = 100 μm. $*p < 0.05$, $**p < 0.01$, $***p < 0.001$. Three independent experiments were performed.

Supplementary Table 1 The primer sequences for experiments

Category	Gene name	Sequence (5'-3')
qRT-PCR	U6 snRNA	F: CTCGCTTCGGCAGCACA<br>R: AACGCTTCACGAATTTGCGT
	GAPDH	F: GACCCCTTCATTGACCTCAACTACATG<br>R: GTCCACCACCCTGTTGCTGTAGCC
	tRNAGln	F: GGTTCCATGGTGTAATGGTAGC<br>R: AGGTTCCACCGAGACTCGAACTC
	tRNA-Tyr-GTA	F: CCTTCGATAGCTCAGCTGGT<br>R: TCCTTCGAGCCGGAATCGAA
	METTL1	F: GCACCTTCCTAACTTCTTCTACAA<br>R: TAGCTCCAGCACATCGGTTA
	WDR4	F: CTGTTAGATGTGGCTGTGAGTC<br>R: GAGTTGGCACCACGGAGAT
	HMGA2	F: ACCCAGGGGAAGACCCAAA<br>R: CCTCTTGGCCGTTTTTCTCCA
Northern blot	NC probe	CCTTCGAT/+A/GC/+T/CAG -Bio*
	Probe	CTGAG/+C/TA/+T/CGAAGG -Bio*
FISH	Probe	GCTCTACCAGCTGAGCTATCGArArG/+G/ -Cy3*
mimic	tRF-5001b	CCTTCGATAGCTCAGCTGGTAGAGC
Inhibitor	tRF-5001b	GCTCTACCAGCTGAGCTATCGAAGG
RNA pull down	NC probe	Bio- CCTTCGAT/+A/GC/+T/CA/+G/CTGGTAGAGC*
	Probe	Bio- GCTCTAC/+C/AG/+C/TG/+A/GCTATCGAAGG*
	Antisense Template	GTAAAACGACGGCCAGTGAGCGCGTAGGGATA<br>ACAGGGTAATGCGCGCGTAATACGACTCACTAT<br>AGGGGCTCTACCAGCTGAGCTATCGAAGGAAAA<br>AAAAAGTCATAGCTGTTTCCTG
	Sense Template	GTAAAACGACGGCCAGTGAGCGCGTAGGGATA<br>ACAGGGTAATGCGCGCGTAATACGACTCACTAT<br>AGGGCCTTCGATAGCTCAGCTGGTAGAGCAAAA<br>AAAAAGTCATAGCTGTTTCCTG

* indicates the presence of modified bases; /+X/, locked nucleic acid modification; X_r: ribonucleotide.
